# Crystal structure and Hirshfeld surface analysis of bis­(μ-4-*tert*-but­oxy-4-oxobut-2-en-2-olato)bis­[(4-*tert*-but­oxy-4-oxobut-2-en-2-olato)ethano­lzinc(II)]

**DOI:** 10.1107/S2056989023003377

**Published:** 2023-04-21

**Authors:** Olherd O. Shtokvysh, Viktoriya V. Dyakonenko, Lyudmila I. Koval, Vasyl I. Pekhnyo

**Affiliations:** a V. I. Vernadskii Institute of General and Inorganic Chemistry, National Academy of Sciences of Ukraine, Akad. Palladin Ave 32/34, Kyiv 03142, Ukraine; b SSI "Institute for Single Crystals" National Academy of Sciences of Ukraine, Nauki Ave 60, Kharkiv 61001, Ukraine; Universität Greifswald, Germany

**Keywords:** crystal structure, complex, zinc, *tert*-butyl aceto­acetate, keto ester, ethanol, binuclear structure

## Abstract

The mol­ecular and crystal structure of the title binuclear Zn^2+^ complex, [Zn_2_(C_8_H_13_O_3_)_4_(C_2_H_5_OH)_2_], with enolated anionic *tert*-butyl­aceto­acetate and ethanol was analysed. The coordination polyhedra of the Zn atoms are distorted octa­hedra formed by six oxygen atoms that belong to three ligand mol­ecules and a coordinated ethanol mol­ecule. In the crystal phase, alternating layers can be distinguished parallel to the *ac* plane. A Hirshfeld surface analysis showed that there are no strong inter­molecular inter­actions in the structure. The most significant contributions to the overall crystal packing are from H⋯H inter­molecular contacts.

## Abstract 2

The crystal structure of bis­(μ-4-*tert*-but­oxy-4-oxobut-2-en-2-olato)bis­[(4-*tert*-but­oxy-4-oxobut-2-en-2-olato)ethano­lzinc(II)] is reported and discussed.

## 1. Chemical context

Metal complexes with β-dicarbonyl ligands are widely used for obtaining metal oxides and, less often, metal films by the metal–organic chemical vapor deposition (MOCVD) process and its variations (Wei *et al.*, 2014 ▸; Han *et al.*, 2017 ▸, 2018 ▸; Nayak *et al.*, 2007 ▸; Cosham *et al.*, 2017 ▸; Kawazoe *et al.*, 2006 ▸; Kamata *et al.*, 1994 ▸), for the catalysis of reduction, oxidation, and oligomerization of unsaturated compounds and cross-coupling reacti& Nobile *et al.*, 1994 ▸). They also exhibit anti­viral activity (Sechi *et al.*, 2006 ▸), in which inter­est has increased significantly in recent years. In addition, β-dicarbonyl complexes of zinc are studied as luminescent materials and anti­oxidants (Aliaga-Alcalde *et al.*, 2012 ▸; Nie *et al.*, 2014 ▸; Turra *et al.*, 2010 ▸). 
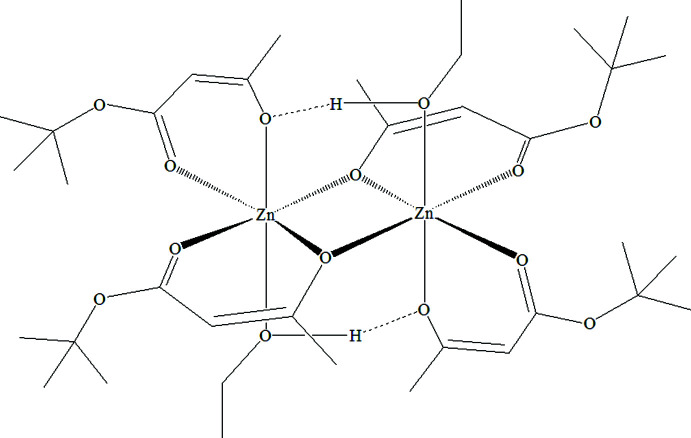


Our research group is developing coordination compounds soluble in non-polar organic solvents, including metal complexes of aceto­acetic acid esters (Koval *et al.*, 2009 ▸), which can potentially be used as environmentally friendly additives to industrial products. Previously, we reported the structure of a trimeric zinc complex synthesized in a rather complicated way using diethyl zinc (Shtokvish *et al.*, 2014 ▸). After that, we developed a much simpler and relatively more efficient method for the synthesis of cobalt and nickel ketoesterates (Shtokvish *et al.*, 2015 ▸, 2017 ▸, Shtokvysh *et al.*, 2018 ▸). The use of this method for the synthesis of Zn complexes made it possible to obtain dimeric complexes with cyclo­hexyl­aceto­acetate (Shtokvysh *et al.*, 2020 ▸) and *tert*-butyl­aceto­acetate. In the present work, we report the synthesis and structural analysis of the new complex [Zn_2_(C_8_H_10_O_3_)_4_(C_2_H_5_OH)_2_].

## 2. Structural commentary

**Figure 1:**
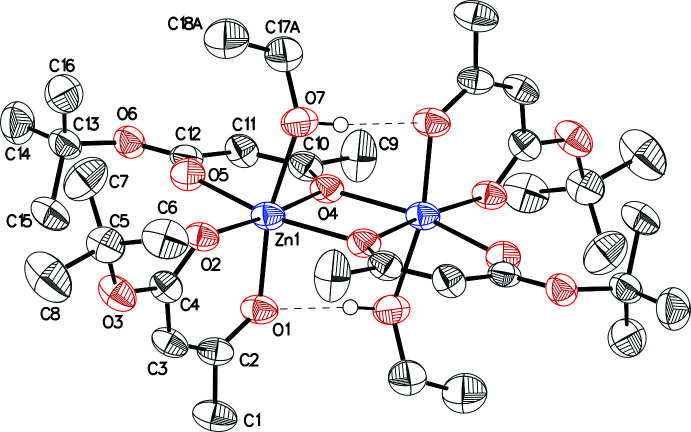
The mol­ecular structure of the title compound, showing 30% probability displacement ellipsoids. H atoms and the minor occupancy disordered component have been omitted for clarity. Unlabelled atoms are related by the symmetry operation 1 − *x*, 1 − *y*, 1 − *z*.

The title compound, systematic name bis­(μ-4-*tert*-but­oxy-4-oxobut-2-en-2-olato)bis­[(4-*tert*-but­oxy-4-oxobut-2-en-2-ol­ato)ethano­lzinc(II)], is a binuclear complex that resides on a special position with the unit cell’s central inversion centre being close to the refined zinc(II) atom and directly in between this and the symmetry-generated zinc atom [symmetry code: (i) −*x* + 1, −*y* + 1, −*z* + 1] (Fig. 1 ▸). The coordination polyhedron of the Zn centre is a distorted octa­hedron formed by six oxygen atoms. One bidentate acetyl­acetonate type ligand (O1, O2) binds only to one zinc centre. Its oxygen atoms occupy an axial (O1) and an equatorial position (O2). The second bidentate ligand (O4, O5) binds the zinc centre only equatorially, while O4 also binds the symmetry-generated second zinc atom of the binuclear complex. This also means that the symmetry-generated O4^i^ atom occupies the fourth equatorial position. The octa­hedral coordination sphere is completed by axially coordinated ethanol (O7). The bonds of zinc atoms with the enol atom of the bridging ligand are not equivalent. The Zn1—O4 bond length in the chelate is shorter than the Zn1—O4^i^ bond length with the symmetry-generated bridging ligand [2.076 (2) and 2.141 (3) Å, respectively; Table 1 ▸]. The Zn—O bond lengths of terminal ligands (O1, O2) are shorter than the Zn—O bonds of bridging ligands (O4, O5) with ranges of 2.031 (3) to 2.039 (3) and of 2.072 (3) to 2.076 (2) Å, respectively (Table 1 ▸). The Zn1—O7 bond length (the bond between the zinc atom and the oxygen of the coordinated ethanol mol­ecule) is the longest in the coordination polyhedron at 2.201 (3) Å (Table 1 ▸). The values of the O—Zn—O bond angles lie in the range 85.30 (11) to 97.46 (12)° (Table 1 ▸). The connection between the nuclei of the complex is additionally stabilized by two intra­molecular hydrogen bonds between the hydrogen atoms of the hydroxyl groups of ethanol and the enol oxygen atoms of the terminal ligands belonging to another nucleus (Table 2 ▸).

## 3. Supra­molecular features

**Figure 2:**
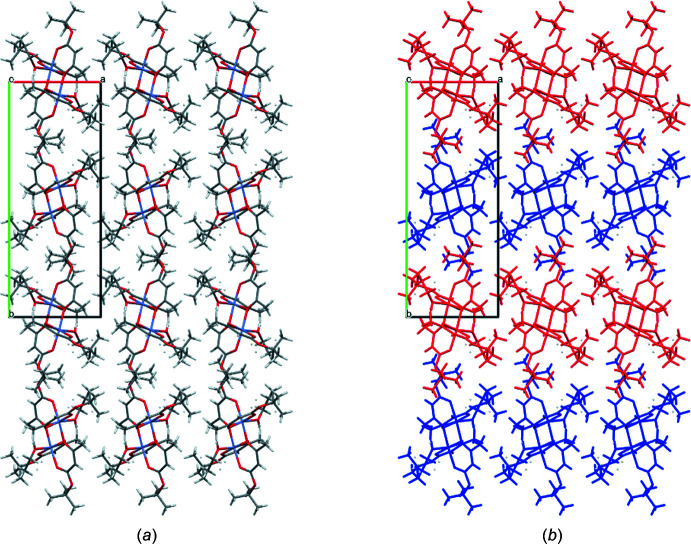
(*a*) Crystal packing of the title compound and (*b*) differently-coloured layers in the same projection.

There are no short inter­molecular contacts between neighbouring mol­ecules in the crystal phase. However, visually we can distinguish alternating layers parallel to the *ac* plane (Fig. 2 ▸ *a*). Mol­ecules in the layer are oriented identically with respect to each other and mirrored with respect to the mol­ecules of the neighbouring layer (Fig. 2 ▸ *b*).

## 4. Hirshfeld surface analysis and finger print plots

**Figure 3:**
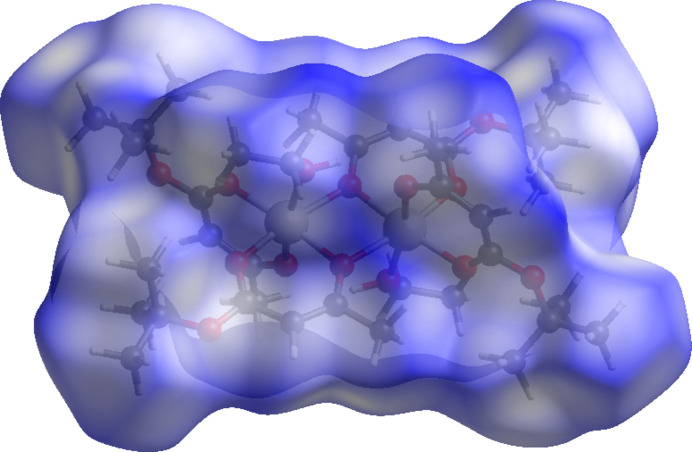
A projection of *d*_norm_ mapped on the Hirshfeld surface, showing the inter­molecular inter­actions within the mol­ecule.

A Hirshfeld surface analysis was performed and the associated two-dimensional fingerprint plots were generated using *Crystal Explorer 21.5* software (Spackman *et al.*, 2021 ▸), with a standard resolution of the three-dimensional *d*_norm_ surfaces plotted over a fixed colour scale of 0.0290 (white) to 1.706 (blue) a.u. (Fig. 3 ▸). Usually contacts shorter than the sums of van der Waals radii are shown in red, those longer in blue, and those approximately equal as white spots. There are no red spots on the *d*_norm_ surface. This indicates that there are no strong inter­molecular inter­actions in the structure.

**Figure 4:**
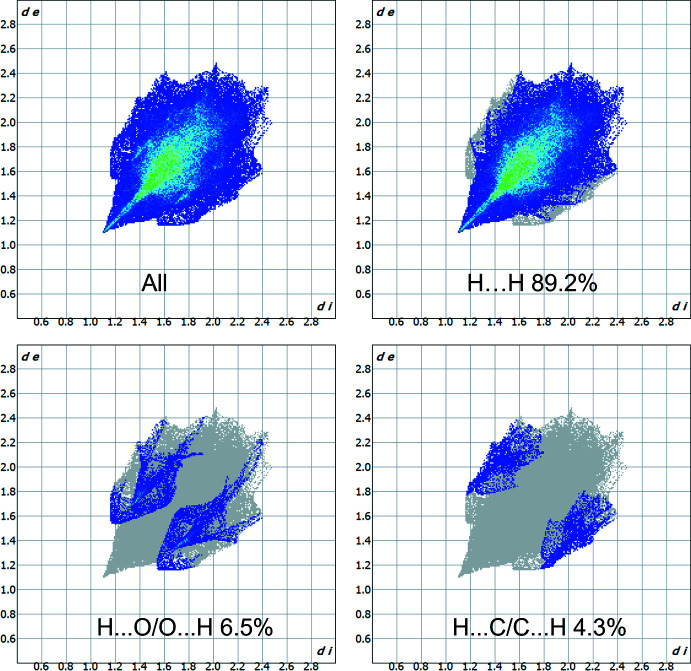
The overall two-dimensional fingerprint plot and those delineated into specified inter­actions.

The overall two-dimensional fingerprint plot, and those decomposed into various inter­actions are given Fig. 4 ▸. The most significant contributions to the overall crystal packing are from H⋯H (89.2%) proximities, which are located mostly in the middle region of the fingerprint plot. There is also a small contribution from H⋯O/O⋯H (6.5%) and H⋯C/C⋯H (4.3%) inter­molecular ‘contacts’.

## 5. Database survey

A search of the Cambridge Structural Database (CSD, Version 5.43, update November 2022; Groom *et al.*, 2016 ▸; ConQuest 2022.3.0; Bruno *et al.*, 2002 ▸) for binuclear complexes with different aceto­acetic esters yielded seven structures that are very similar to the title compound. Among these structures are three structures with the metal being cobalt (refcodes BENNUG, BENPAO; Shtokvish *et al.*, 2017 ▸; WARHAB; Shtokvish *et al.*, 2015 ▸), three structures with nickel (refcodes WOCXOE, WOCXUK, WOCYAR; Shtokvysh *et al.*, 2018 ▸) and one with zinc (refcode GARBOU; Shtokvysh *et al.*, 2020 ▸). The coordination centres in all cases have an octa­hedral geometric environment. The *M*—O bond lengths (1.997 to 2.082 Å) are consistently shorter in the terminal ligand than the *M*—O bond lengths (2.088 to 2.184 Å) of the bridging ligands.

## 6. Synthesis and crystallization

The title compound was synthesized in accordance with the methodology reported earlier (Shtokvish *et al.*, 2015 ▸). ZnCl_2_ (0.1 g, 7 mmol) was dissolved in 2 ml of ethanol (azeotrope with water, 95.6% alcohol). Then liquid *tert-*butyl aceto­acetate was added to the solution (0.244 ml, 14 mmol). The components were then mixed. The test tube with the reaction mixture was placed in a container together with a vessel containing tri­ethyl­amine (0.4 ml, 28 mmol). The container was sealed and left in the refrigerator for 1–2 days at a temperature of 281 K. The structural study was performed for a crystal taken directly and immediately from the reaction mixture, since this compound is prone to degradeation. The crystals were filtered on a P2 (P100) fritted glass filter (to separate thin powders of by-products and degradeation products), then washed several times with ethanol and dried in air for no more than 1 h. The yield is 0.078 g, which is 25.3% of the theoretical value. The obtained crystals can be stored at 261 K and below.

## 7. Refinement

Crystal data, data collection and structure refinement details are summarized in Table 3 ▸. H atoms were placed in calculated positions [C—H = 0.93 Å (0.96 Å for C-meth­yl)] and refined as riding with *U*_iso_(H) = 1.2*U*_eq_(C) or 1.5*U*_eq_(C-meth­yl).

The C atoms of the coordinated ethanol mol­ecule are disordered over two positions with an occupancy of 50%. Restraints were applied to the bond lengths in the disordered parts (O—C = 1.45 Å, C—C = 1.49 Å) within a standard deviation of 0.02 Å. The position of the O-bound hydrogen atom was determined from the electron-density map. The O-bound hydrogen atom was refined freely with full occupancy restraining only the O—H bond length to 0.86 Å within a standard deviation of 0.02 Å.

## Supplementary Material

Crystal structure: contains datablock(s) global, I. DOI: 10.1107/S2056989023003377/yz2031sup1.cif

Structure factors: contains datablock(s) I. DOI: 10.1107/S2056989023003377/yz2031Isup2.hkl

CCDC reference: 2255847

Additional supporting information:  crystallographic information; 3D view; checkCIF report

## Figures and Tables

**Table 1:** Selected geometric parameters (Å, °)

Zn1—O1	2.031 (3)	Zn1—O4	2.076 (2)
Zn1—O2	2.039 (3)	Zn1—O5	2.072 (3)
Zn1—O4^i^	2.141 (3)	Zn1—O7	2.201 (3)
			
O1—Zn1—O2	90.99 (11)	O2—Zn1—O7	93.30 (12)
O1—Zn1—O4	90.61 (11)	O4—Zn1—O7	86.49 (12)
O1—Zn1—O5	97.46 (12)	O5—Zn1—O4	88.08 (11)
O2—Zn1—O5	85.30 (11)	O5—Zn1—O7	94.74 (12)

Symmetry code: (i) 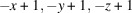 .

**Table 2:** Hydrogen-bond geometry (Å, °)

*D*—H⋯*A*	*D*—H	H⋯*A*	*D*⋯*A*	*D*—H⋯*A*
O7—H7⋯O1^i^	0.86 (1)	2.01 (1)	2.861 (4)	171 (4)

Symmetry code: (i) 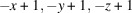 .

**Table 3:** Experimental details

Crystal data	
Chemical formula	[Zn_2_(C_8_H_13_O_3_)_4_(C_2_H_6_O)_2_]
*M*_r_	851.61
Crystal system, space group	Monoclinic, *P*2_1_/*c*
Temperature (K)	295
*a*, *b*, *c* (Å)	9.1689 (5), 22.8882 (10), 11.0743 (5)
β (°)	103.043 (5)
*V* (Å^3^)	2264.10 (19)
*Z*	2
Radiation type	Mo *K*α
μ (mm^−1^)	1.12
Crystal size (mm)	0.5 × 0.4 × 0.2

Data collection	
Diffractometer	Xcalibur, Sapphire3
Absorption correction	Multi-scan (*CrysAlis PRO*; Rigaku OD, 2018 ▸)
*T*_min_, *T*_max_	0.460, 1.000
No. of measured, independent and observed [*I* > 2σ(*I*)] reflections	18037, 4622, 3257
*R*_int_	0.051
(sin θ/λ)_max_ (Å^−1^)	0.625

Refinement	
*R*[*F* ^2^ > 2σ(*F* ^2^)], *wR*(*F* ^2^), *S*	0.061, 0.154, 1.09
No. of reflections	4622
No. of parameters	268
No. of restraints	53
H-atom treatment	H atoms treated by a mixture of independent and constrained refinement
Δρ_max_, Δρ_min_ (e Å^−3^)	0.56, −0.34

Computer programs: *CrysAlis PRO* (Rigaku OD, 2018 ▸), *OLEX2.solve* (Bourhis *et al.*, 2015 ▸), *SHELXL2019/3* (Sheldrick, 2015 ▸), *OLEX2*(Dolomanov *et al.*, 2009 ▸) and *Mercury* (Macrae *et al.*, 2020 ▸).

## supplementary crystallographic information

### Crystal data

**Table:**

[Zn_2_(C_8_H_13_O_3_)_4_(C_2_H_6_O)_2_]	*F*(000) = 904
*M**_r_* = 851.61	*D*_x_ = 1.249 Mg m^−^^3^
Monoclinic, *P*2_1_/*c*	Mo *K*α radiation, λ = 0.71073 Å
*a* = 9.1689 (5) Å	Cell parameters from 2231 reflections
*b* = 22.8882 (10) Å	θ = 3.7–21.8°
*c* = 11.0743 (5) Å	µ = 1.12 mm^−^^1^
β = 103.043 (5)°	*T* = 295 K
*V* = 2264.10 (19) Å^3^	Block, colourless
*Z* = 2	0.5 × 0.4 × 0.2 mm

### Data collection

**Table:**

Xcalibur, Sapphire3 diffractometer	3257 reflections with *I* > 2σ(*I*)
Detector resolution: 16.1827 pixels mm^-1^	*R*_int_ = 0.051
ω scans	θ_max_ = 26.4°, θ_min_ = 3.2°
Absorption correction: multi-scan (CrysAlisPro; Rigaku OD, 2018)	*h* = −11→11
*T*_min_ = 0.460, *T*_max_ = 1.000	*k* = −28→28
18037 measured reflections	*l* = −8→13
4622 independent reflections	

### Refinement

**Table:**

Refinement on *F*^2^	Primary atom site location: iterative
Least-squares matrix: full	Hydrogen site location: mixed
*R*[*F*^2^ > 2σ(*F*^2^)] = 0.061	H atoms treated by a mixture of independent and constrained refinement
*wR*(*F*^2^) = 0.154	*w* = 1/[σ^2^(*F*_o_^2^) + (0.0617*P*)^2^ + 0.6818*P*] where *P* = (*F*_o_^2^ + 2*F*_c_^2^)/3
*S* = 1.09	(Δ/σ)_max_ < 0.001
4622 reflections	Δρ_max_ = 0.56 e Å^−^^3^
268 parameters	Δρ_min_ = −0.34 e Å^−^^3^
53 restraints	

### Special details

**Table:**

Geometry. All esds (except the esd in the dihedral angle between two l.s. planes) are estimated using the full covariance matrix. The cell esds are taken into account individually in the estimation of esds in distances, angles and torsion angles; correlations between esds in cell parameters are only used when they are defined by crystal symmetry. An approximate (isotropic) treatment of cell esds is used for estimating esds involving l.s. planes.

### Fractional atomic coordinates and isotropic or equivalent isotropic displacement parameters (Å^2^)

**Table:**

	*x*	*y*	*z*	*U*_iso_*/*U*_eq_	Occ. (<1)
Zn1	0.54544 (5)	0.44504 (2)	0.42445 (4)	0.0662 (2)	
O1	0.3922 (3)	0.47158 (13)	0.2720 (3)	0.0764 (8)	
O2	0.6833 (3)	0.41548 (12)	0.3169 (2)	0.0671 (7)	
O3	0.7330 (3)	0.38286 (15)	0.1401 (3)	0.0882 (9)	
O4	0.3987 (3)	0.46505 (12)	0.5364 (2)	0.0672 (7)	
O5	0.4756 (3)	0.35898 (12)	0.4248 (3)	0.0772 (8)	
O6	0.3134 (3)	0.28661 (12)	0.4379 (2)	0.0738 (7)	
O7	0.7190 (4)	0.43704 (14)	0.5970 (3)	0.0911 (9)	
C1	0.2636 (5)	0.4793 (2)	0.0629 (4)	0.0938 (15)	
H1A	0.172653	0.468769	0.086530	0.141*	
H1B	0.266070	0.461221	−0.014835	0.141*	
H1C	0.267881	0.521015	0.054530	0.141*	
C2	0.3957 (4)	0.45897 (18)	0.1606 (4)	0.0672 (10)	
C3	0.5095 (4)	0.42909 (19)	0.1243 (4)	0.0706 (11)	
H3	0.496600	0.421072	0.040242	0.085*	
C4	0.6431 (4)	0.40970 (17)	0.2033 (4)	0.0666 (10)	
C5	0.8841 (5)	0.3624 (2)	0.1982 (5)	0.0897 (14)	
C6	0.9817 (5)	0.4121 (3)	0.2556 (6)	0.1108 (18)	
H6A	0.963480	0.445332	0.201407	0.166*	
H6B	1.084927	0.400825	0.268616	0.166*	
H6C	0.959237	0.422025	0.333682	0.166*	
C7	0.8762 (7)	0.3149 (3)	0.2906 (7)	0.131 (2)	
H7A	0.845972	0.331407	0.360811	0.197*	
H7B	0.972864	0.297042	0.317296	0.197*	
H7C	0.804662	0.285930	0.252621	0.197*	
C8	0.9363 (7)	0.3385 (4)	0.0867 (6)	0.155 (3)	
H8A	0.862961	0.311646	0.042344	0.233*	
H8B	1.030025	0.318583	0.114494	0.233*	
H8C	0.948707	0.370145	0.033114	0.233*	
C9	0.1984 (7)	0.4577 (2)	0.6368 (6)	0.123 (2)	
H9A	0.256051	0.467295	0.717969	0.184*	
H9B	0.120541	0.430766	0.643732	0.184*	
H9C	0.154656	0.492652	0.596048	0.184*	
C10	0.2986 (5)	0.43028 (19)	0.5624 (4)	0.0705 (11)	
C11	0.2793 (5)	0.37331 (19)	0.5304 (4)	0.0739 (11)	
H11	0.203184	0.353451	0.556179	0.089*	
C12	0.3643 (5)	0.34110 (17)	0.4612 (3)	0.0652 (10)	
C13	0.3831 (5)	0.24431 (19)	0.3696 (4)	0.0776 (12)	
C14	0.2860 (7)	0.1908 (2)	0.3683 (5)	0.1141 (19)	
H14A	0.285700	0.179792	0.451860	0.171*	
H14B	0.325115	0.159240	0.327984	0.171*	
H14C	0.185651	0.199405	0.324153	0.171*	
C15	0.3773 (8)	0.2664 (3)	0.2393 (4)	0.122 (2)	
H15A	0.275191	0.274108	0.198153	0.183*	
H15B	0.418317	0.237424	0.193851	0.183*	
H15C	0.434563	0.301784	0.243508	0.183*	
C16	0.5409 (6)	0.2315 (2)	0.4396 (5)	0.1051 (17)	
H16A	0.601067	0.265992	0.441878	0.158*	
H16B	0.581885	0.200646	0.398803	0.158*	
H16C	0.540080	0.219781	0.522665	0.158*	
C17A	0.7360 (17)	0.3935 (4)	0.6946 (9)	0.120 (2)	0.502 (9)
H17A	0.640986	0.375180	0.695965	0.144*	0.502 (9)
H17B	0.777579	0.410583	0.775206	0.144*	0.502 (9)
C17B	0.7398 (17)	0.3790 (2)	0.6508 (10)	0.119 (2)	0.498 (9)
H17C	0.758504	0.352034	0.588523	0.142*	0.498 (9)
H17D	0.647661	0.367100	0.672769	0.142*	0.498 (9)
C18A	0.8418 (16)	0.3510 (6)	0.6591 (14)	0.151 (3)	0.502 (9)
H18A	0.939453	0.368299	0.671692	0.226*	0.502 (9)
H18B	0.807363	0.340685	0.573319	0.226*	0.502 (9)
H18C	0.846888	0.316516	0.709264	0.226*	0.502 (9)
C18B	0.8644 (15)	0.3742 (6)	0.7627 (12)	0.142 (3)	0.498 (9)
H18D	0.941201	0.349365	0.744778	0.212*	0.498 (9)
H18E	0.827299	0.357902	0.829736	0.212*	0.498 (9)
H18F	0.905194	0.412323	0.785632	0.212*	0.498 (9)
H7	0.692 (4)	0.4672 (10)	0.633 (3)	0.079 (14)*	

### Atomic displacement parameters (Å^2^)

**Table:**

	*U*^11^	*U*^22^	*U*^33^	*U*^12^	*U*^13^	*U*^23^
Zn1	0.0695 (3)	0.0780 (3)	0.0560 (3)	0.0014 (2)	0.0247 (2)	0.0122 (2)
O1	0.0709 (18)	0.101 (2)	0.0607 (17)	0.0149 (15)	0.0226 (14)	0.0153 (15)
O2	0.0607 (15)	0.0879 (18)	0.0556 (16)	0.0049 (13)	0.0193 (13)	0.0093 (14)
O3	0.0650 (18)	0.133 (3)	0.0683 (19)	0.0093 (17)	0.0196 (15)	−0.0132 (18)
O4	0.0752 (17)	0.0740 (16)	0.0594 (16)	−0.0039 (14)	0.0297 (14)	0.0126 (13)
O5	0.0866 (19)	0.0788 (18)	0.0735 (19)	−0.0078 (16)	0.0333 (16)	0.0084 (14)
O6	0.0857 (19)	0.0751 (18)	0.0636 (17)	−0.0105 (15)	0.0233 (15)	−0.0032 (14)
O7	0.110 (2)	0.090 (2)	0.073 (2)	0.0219 (18)	0.0186 (18)	0.0182 (16)
C1	0.067 (3)	0.141 (4)	0.072 (3)	0.006 (3)	0.012 (2)	0.020 (3)
C2	0.058 (2)	0.086 (3)	0.059 (2)	−0.008 (2)	0.015 (2)	0.016 (2)
C3	0.058 (2)	0.106 (3)	0.050 (2)	−0.004 (2)	0.0160 (19)	0.002 (2)
C4	0.062 (2)	0.081 (3)	0.063 (3)	−0.009 (2)	0.026 (2)	−0.001 (2)
C5	0.067 (3)	0.120 (4)	0.085 (3)	0.015 (3)	0.023 (3)	−0.001 (3)
C6	0.063 (3)	0.155 (5)	0.113 (4)	−0.004 (3)	0.019 (3)	0.009 (4)
C7	0.114 (5)	0.112 (4)	0.171 (7)	0.034 (4)	0.039 (5)	0.020 (4)
C8	0.090 (4)	0.257 (9)	0.123 (5)	0.050 (5)	0.032 (4)	−0.049 (5)
C9	0.135 (5)	0.100 (4)	0.169 (6)	−0.036 (3)	0.108 (5)	−0.027 (4)
C10	0.074 (3)	0.086 (3)	0.058 (2)	−0.010 (2)	0.027 (2)	0.008 (2)
C11	0.078 (3)	0.084 (3)	0.067 (3)	−0.020 (2)	0.031 (2)	−0.002 (2)
C12	0.075 (3)	0.073 (3)	0.046 (2)	−0.003 (2)	0.0083 (19)	0.0114 (18)
C13	0.097 (3)	0.084 (3)	0.053 (2)	−0.007 (3)	0.020 (2)	−0.001 (2)
C14	0.144 (5)	0.089 (3)	0.113 (4)	−0.024 (3)	0.037 (4)	−0.018 (3)
C15	0.186 (6)	0.125 (4)	0.056 (3)	0.002 (4)	0.030 (4)	−0.001 (3)
C16	0.109 (4)	0.107 (4)	0.103 (4)	0.005 (3)	0.032 (4)	−0.001 (3)
C17A	0.140 (4)	0.111 (4)	0.095 (4)	0.033 (4)	−0.005 (4)	−0.002 (3)
C17B	0.138 (4)	0.109 (4)	0.095 (4)	0.038 (4)	−0.003 (4)	0.000 (3)
C18A	0.159 (6)	0.139 (6)	0.132 (6)	0.027 (6)	−0.014 (6)	0.004 (6)
C18B	0.157 (6)	0.135 (6)	0.114 (6)	0.032 (5)	−0.008 (6)	0.006 (5)

### Geometric parameters (Å, º)

**Table:**

Zn1—O1	2.031 (3)	C8—H8B	0.9600
Zn1—O2	2.039 (3)	C8—H8C	0.9600
Zn1—O4^i^	2.141 (3)	C9—H9A	0.9600
Zn1—O4	2.076 (2)	C9—H9B	0.9600
Zn1—O5	2.072 (3)	C9—H9C	0.9600
Zn1—O7	2.201 (3)	C9—C10	1.503 (6)
O1—C2	1.273 (5)	C10—C11	1.352 (6)
O2—C4	1.235 (5)	C11—H11	0.9300
O3—C4	1.345 (5)	C11—C12	1.417 (6)
O3—C5	1.467 (5)	C13—C14	1.512 (6)
O4—C10	1.295 (4)	C13—C15	1.519 (6)
O5—C12	1.248 (5)	C13—C16	1.508 (6)
O6—C12	1.336 (5)	C14—H14A	0.9600
O6—C13	1.461 (5)	C14—H14B	0.9600
O7—C17A	1.452 (2)	C14—H14C	0.9600
O7—C17B	1.450 (2)	C15—H15A	0.9600
O7—H7	0.860 (2)	C15—H15B	0.9600
C1—H1A	0.9600	C15—H15C	0.9600
C1—H1B	0.9600	C16—H16A	0.9600
C1—H1C	0.9600	C16—H16B	0.9600
C1—C2	1.505 (6)	C16—H16C	0.9600
C2—C3	1.381 (5)	C17A—H17A	0.9700
C3—H3	0.9300	C17A—H17B	0.9700
C3—C4	1.407 (6)	C17A—C18A	1.489 (2)
C5—C6	1.498 (7)	C17B—H17C	0.9700
C5—C7	1.506 (7)	C17B—H17D	0.9700
C5—C8	1.522 (7)	C17B—C18B	1.487 (2)
C6—H6A	0.9600	C18A—H18A	0.9600
C6—H6B	0.9600	C18A—H18B	0.9600
C6—H6C	0.9600	C18A—H18C	0.9600
C7—H7A	0.9600	C18B—H18D	0.9600
C7—H7B	0.9600	C18B—H18E	0.9600
C7—H7C	0.9600	C18B—H18F	0.9600
C8—H8A	0.9600		
			
O1—Zn1—O2	90.99 (11)	H8A—C8—H8C	109.5
O1—Zn1—O4^i^	88.27 (12)	H8B—C8—H8C	109.5
O1—Zn1—O4	90.61 (11)	H9A—C9—H9B	109.5
O1—Zn1—O5	97.46 (12)	H9A—C9—H9C	109.5
O1—Zn1—O7	167.36 (12)	H9B—C9—H9C	109.5
O2—Zn1—O4	173.35 (10)	C10—C9—H9A	109.5
O2—Zn1—O4^i^	106.60 (10)	C10—C9—H9B	109.5
O2—Zn1—O5	85.30 (11)	C10—C9—H9C	109.5
O2—Zn1—O7	93.30 (12)	O4—C10—C9	114.6 (4)
O4—Zn1—O4^i^	79.90 (10)	O4—C10—C11	126.3 (4)
O4^i^—Zn1—O7	79.11 (11)	C11—C10—C9	119.1 (4)
O4—Zn1—O7	86.49 (12)	C10—C11—H11	117.0
O5—Zn1—O4	88.08 (11)	C10—C11—C12	126.0 (4)
O5—Zn1—O4^i^	166.76 (10)	C12—C11—H11	117.0
O5—Zn1—O7	94.74 (12)	O5—C12—O6	121.2 (4)
C2—O1—Zn1	125.0 (3)	O5—C12—C11	126.4 (4)
C4—O2—Zn1	123.2 (3)	O6—C12—C11	112.3 (4)
C4—O3—C5	123.2 (3)	O6—C13—C14	102.5 (4)
Zn1—O4—Zn1^i^	100.10 (10)	O6—C13—C15	110.2 (4)
C10—O4—Zn1^i^	134.0 (3)	O6—C13—C16	110.1 (4)
C10—O4—Zn1	125.8 (3)	C14—C13—C15	111.5 (4)
C12—O5—Zn1	126.0 (3)	C16—C13—C14	110.0 (4)
C12—O6—C13	123.0 (3)	C16—C13—C15	112.2 (4)
Zn1—O7—H7	96 (3)	C13—C14—H14A	109.5
C17A—O7—Zn1	129.7 (7)	C13—C14—H14B	109.5
C17A—O7—H7	102 (3)	C13—C14—H14C	109.5
C17B—O7—Zn1	115.8 (5)	H14A—C14—H14B	109.5
C17B—O7—H7	125 (3)	H14A—C14—H14C	109.5
H1A—C1—H1B	109.5	H14B—C14—H14C	109.5
H1A—C1—H1C	109.5	C13—C15—H15A	109.5
H1B—C1—H1C	109.5	C13—C15—H15B	109.5
C2—C1—H1A	109.5	C13—C15—H15C	109.5
C2—C1—H1B	109.5	H15A—C15—H15B	109.5
C2—C1—H1C	109.5	H15A—C15—H15C	109.5
O1—C2—C1	115.7 (4)	H15B—C15—H15C	109.5
O1—C2—C3	125.4 (4)	C13—C16—H16A	109.5
C3—C2—C1	118.8 (4)	C13—C16—H16B	109.5
C2—C3—H3	117.1	C13—C16—H16C	109.5
C2—C3—C4	125.7 (4)	H16A—C16—H16B	109.5
C4—C3—H3	117.1	H16A—C16—H16C	109.5
O2—C4—O3	120.1 (4)	H16B—C16—H16C	109.5
O2—C4—C3	128.1 (4)	O7—C17A—H17A	111.2
O3—C4—C3	111.7 (4)	O7—C17A—H17B	111.2
O3—C5—C6	111.0 (4)	O7—C17A—C18A	102.6 (8)
O3—C5—C7	110.1 (4)	H17A—C17A—H17B	109.2
O3—C5—C8	101.4 (4)	C18A—C17A—H17A	111.2
C6—C5—C7	112.0 (5)	C18A—C17A—H17B	111.2
C6—C5—C8	110.5 (5)	O7—C17B—H17C	108.6
C7—C5—C8	111.4 (5)	O7—C17B—H17D	108.6
C5—C6—H6A	109.5	O7—C17B—C18B	114.5 (8)
C5—C6—H6B	109.5	H17C—C17B—H17D	107.6
C5—C6—H6C	109.5	C18B—C17B—H17C	108.6
H6A—C6—H6B	109.5	C18B—C17B—H17D	108.6
H6A—C6—H6C	109.5	C17A—C18A—H18A	109.5
H6B—C6—H6C	109.5	C17A—C18A—H18B	109.5
C5—C7—H7A	109.5	C17A—C18A—H18C	109.5
C5—C7—H7B	109.5	H18A—C18A—H18B	109.5
C5—C7—H7C	109.5	H18A—C18A—H18C	109.5
H7A—C7—H7B	109.5	H18B—C18A—H18C	109.5
H7A—C7—H7C	109.5	C17B—C18B—H18D	109.5
H7B—C7—H7C	109.5	C17B—C18B—H18E	109.5
C5—C8—H8A	109.5	C17B—C18B—H18F	109.5
C5—C8—H8B	109.5	H18D—C18B—H18E	109.5
C5—C8—H8C	109.5	H18D—C18B—H18F	109.5
H8A—C8—H8B	109.5	H18E—C18B—H18F	109.5
			
Zn1—O1—C2—C1	175.2 (3)	C2—C3—C4—O2	0.0 (7)
Zn1—O1—C2—C3	−5.2 (6)	C2—C3—C4—O3	−178.9 (4)
Zn1—O2—C4—O3	−171.2 (3)	C4—O3—C5—C6	−60.4 (6)
Zn1—O2—C4—C3	10.0 (6)	C4—O3—C5—C7	64.2 (6)
Zn1^i^—O4—C10—C9	−0.3 (6)	C4—O3—C5—C8	−177.8 (5)
Zn1—O4—C10—C9	−174.9 (4)	C5—O3—C4—O2	−3.7 (6)
Zn1^i^—O4—C10—C11	−179.5 (3)	C5—O3—C4—C3	175.2 (4)
Zn1—O4—C10—C11	5.9 (6)	C9—C10—C11—C12	−179.8 (5)
Zn1—O5—C12—O6	168.2 (3)	C10—C11—C12—O5	4.4 (7)
Zn1—O5—C12—C11	−12.6 (6)	C10—C11—C12—O6	−176.3 (4)
Zn1—O7—C17A—C18A	95.5 (12)	C12—O6—C13—C14	−180.0 (4)
Zn1—O7—C17B—C18B	176.2 (11)	C12—O6—C13—C15	−61.2 (5)
O1—C2—C3—C4	−2.8 (7)	C12—O6—C13—C16	63.0 (5)
O4—C10—C11—C12	−0.6 (8)	C13—O6—C12—O5	0.2 (6)
C1—C2—C3—C4	176.8 (4)	C13—O6—C12—C11	−179.1 (4)

Symmetry code: (i) −*x*+1, −*y*+1, −*z*+1.

### Hydrogen-bond geometry (Å, º)

**Table:**

*D*—H···*A*	*D*—H	H···*A*	*D*···*A*	*D*—H···*A*
O7—H7···O1^i^	0.86 (1)	2.01 (1)	2.861 (4)	171 (4)

Symmetry code: (i) −*x*+1, −*y*+1, −*z*+1.

## References

[bb1] Aliaga-Alcalde, N., Rodríguez, L., Ferbinteanu, M., Höfer, P. & Weyhermüller, T. (2012). *Inorg. Chem.* **51**, 864–873. 10.1021/ic201420d 22220749

[bb2] Bourhis, L. J., Dolomanov, O. V., Gildea, R. J., Howard, J. A. K. & Puschmann, H. (2015). *Acta Cryst.* A**71**, 59–75. 10.1107/S2053273314022207 PMC4283469 25537389

[bb3] Bruno, I. J., Cole, J. C., Edgington, P. R., Kessler, M., Macrae, C. F., McCabe, P., Pearson, J. & Taylor, R. (2002). *Acta Cryst.* B**58**, 389–397. 10.1107/s0108768102003324 12037360

[bb4] Cosham, S. D., Richards, S. P., Manning, T., Hill, M. S., Johnson, A. L. & Molloy, K. C. (2017). *Eur. J. Inorg. Chem.* pp. 1868–1876.

[bb5] Dolomanov, O. V., Bourhis, L. J., Gildea, R. J., Howard, J. A. K. & Puschmann, H. (2009). *J. Appl. Cryst.* **42**, 339–341.

[bb7] Groom, C. R., Bruno, I. J., Lightfoot, M. P. & Ward, S. C. (2016). *Acta Cryst.* B**72**, 171–179. 10.1107/S2052520616003954 PMC4822653 27048719

[bb8] Han, H., Wei, Zh., Barry, M. C., Carozza, J. C., Alkan, M., Rogachev, A. Yu., Filatov, A. S., Abakumov, A. M. & Dikarev, E. V. (2018). *Chem. Sci.* **9**, 4736–4745. 10.1039/c8sc00917a PMC5982224 29910924

[bb9] Han, H., Wei, Zh., Barry, M. C., Filatov, A. S. & Dikarev, E. V. (2017). *Dalton Trans.* **46**, 5644–5649. 10.1039/c6dt04602a 28144673

[bb10] Kamata, K., Nishino, J., Ohshio, S., Maruyama, K. & Ohtuki, M. (1994). *J. Am. Ceram. Soc.* **77**, 505–508.

[bb11] Kawazoe, T., Kobayashi, K. & Ohtsu, M. (2006). *Appl. Phys. B*, **84**, 247–251.

[bb12] Koval, L. I., Dzyuba, V. I., Bon, V. V., Ilnitska, O. L. & Pekhnyo, V. I. (2009). *Polyhedron*, **28**, 2698–2702.

[bb13] Macrae, C. F., Sovago, I., Cottrell, S. J., Galek, P. T. A., McCabe, P., Pidcock, E., Platings, M., Shields, G. P., Stevens, J. S., Towler, M. & Wood, P. A. (2020). *J. Appl. Cryst.* **53**, 226–235. 10.1107/S1600576719014092 PMC6998782 32047413

[bb14] Mastrorilli, P. & Nobile, C. F. (1994). *J. Mol. Catal.* **94**, 19–26.

[bb16] Nayak, S. K., Jena, A., Neelgund, G. M., Shivashankar, S. A. & Guru Row, T. N. (2007). *Acta Cryst.* E**63**, m1604.

[bb17] Nie, C., Zhang, Q., Ding, H., Huang, B., Wang, X., Zhao, X., Li, S., Zhou, H., Wu, J. & Tian, Y. (2014). *Dalton Trans.* **43**, 599–608. 10.1039/c3dt51318a 24132227

[bb18] Rigaku OD (2018). *CrysAlis PRO*. Rigaku Oxford Diffraction, Yarnton, England.

[bb19] Sechi, M., Bacchi, A., Carcelli, M., Compari, C., Duce, E., Fisicaro, E., Rogolino, D., Gates, P., Derudas, M., Al-Mawsawi, L. Q. & Neamati, N. (2006). *J. Med. Chem.* **49**, 4248–4260. 10.1021/jm060193m 16821784

[bb20] Sheldrick, G. M. (2015). *Acta Cryst.* C**71**, 3–8.

[bb21] Shtokvish, O. O., Koval, L. I., Dyakonenko, V. V. & Pekhnyo, V. I. (2017). *Ukr. Chem. J.* **83**, 34–37. https://ucj.org.ua/index.php/journal/issue/view/95/5-2017

[bb22] Shtokvish, O. O., Koval, L. I. & Pekhnyo, V. I. (2014). *Acta Cryst.* E**70**, 483–485. 10.1107/S1600536814024337 PMC4257390 25552972

[bb23] Shtokvish, O. O., Koval, L. I. & Pekhnyo, V. I. (2015). *Ukr. Chem. J.* **81**, 92–98. https://ucj.org.ua/index.php/journal/issue/view/43/12-2015

[bb24] Shtokvysh, O., Koval, L., Dyakonenko, V. & Pekhnyo, V. (2020). *Bull. Taras Shevchenko Nat. Univ. Kyiv Chem.* **1**, 66–69. https://doi.org/10.17721/1728-2209.2020.1(57).16

[bb25] Shtokvysh, O. O., Koval, L. I., Dyakonenko, V. V. & Pekhnyo, V. I. (2018). *Ukr. Chem. J.* **84**, 13–19. https://ucj.org.ua/index.php/journal/issue/view/3/3

[bb26] Spackman, P. R., Turner, M. J., McKinnon, J. J., Wolff, S. K., Grimwood, D. J., Jayatilaka, D. & Spackman, M. A. (2021). *J. Appl. Cryst.* **54**, 1006–1011. 10.1107/S1600576721002910 PMC8202033 34188619

[bb27] Turrà, N., Neuenschwander, U., Baiker, A., Peeters, J. & Hermans, I. (2010). *Chem. Eur. J.* **16**, 13226–13235. 10.1002/chem.201000489 20945308

[bb28] Wei, Zh., Han, H., Filatov, A. S. & Dikarev, E. V. (2014). *Chem. Sci.* **5**, 813–818.

